# Materialistic Cues Boosts Personal Relative Deprivation

**DOI:** 10.3389/fpsyg.2016.01236

**Published:** 2016-08-15

**Authors:** Hong Zhang, Wen Zhang

**Affiliations:** Department of Psychology, Nanjing UniversityNanjing, China

**Keywords:** personal relative deprivation, materialistic cues

## Abstract

Three studies investigated whether exposure to materialistic cues would increase perceptions of personal relative deprivation and related emotional reactions. In Study 1, individuals who were surveyed in front of a luxury store reported higher levels of personal relative deprivation than those surveyed in front of an ordinary building. In Study 2, participants who viewed pictures of luxurious goods experienced greater personal relative deprivation than those viewed pictures of neutral scenes. Study 3 replicated the results from Study 2, with a larger sample size and a more refined assessment of relative deprivation. Implications of these findings for future studies on relative deprivation and materialism are discussed.

## Introduction

We encounter materialistic cues on a daily basis. When you walk out, you see shopping malls with grand electronic billboards standing against the sky, and prestige cars roaring past the street. If you turn on the TV or surf the Internet, you see entertainment shows demonstrating upper class lifestyle, and commercials on all kinds of goods urging you to buy. It is no doubt that materialistic signs have a significant impact upon individuals’ psychology and behavior. In recent years we witness an increasing interest into identifying the psychological implications of signs of wealth and material possessions (e.g., Vohs et al., 2006; Dunn and Searle, 2010; Bauer et al., 2012). For instance, monetary and materialistic cues have been found to alter individuals’ self-perceptions (e.g., Ashikali and Dittmar, 2012) and mate preferences (e.g., Yong and Li, 2012). In the current studies, we investigated whether materialistic cues would evoke personal relative deprivation among three Chinese samples.

### Personal Relative Deprivation and Its Preconditions

Following Crosby (1976), we define personal relative deprivation as a sense that one does not get what he/she deserves relative to others, accompanied by feelings of resentment and anger^1^. Rather than a consequence of absolute inadequacy of economic resources, personal relative deprivation is evoked by the perception that one is unjustly worth-off than others. Relative deprivation is an important yet somewhat understudied concept in social psychology. It has been linked to a variety of consequences, ranging from physical health and psychological well-being, to inter-group attitudes and collective action (see Smith et al., 2012, for a review). We deem it to be understudied because compared to related variables, such as belief in a just world (e.g., Lerner, 1980), or system justification (e.g., Jost et al., 2004), relative deprivation has drawn relatively less research attention from psychologists. This is partially due to the fact that there has been much controversy over the conceptualization and measure of relative deprivation. For instance, whereas some studies showed that feelings such as resentment, anger or dissatisfaction, are crucial for behavioral responses to occur (e.g., Guimond and Dubé-Simard, 1983), many early studies focused only on the cognitive component of relative deprivation (e.g., Pettigrew and Meertens, 1995).

The preconditions of relative deprivation, too, are diverse from each other as identified by different researchers. Bernstein and Crosby (1980) have summarized four sets of preconditions of relative deprivation. Specifically, Davis (1959) suggested that relative deprivation would be experienced if individuals (i) want X, (ii) compare themselves to similar others who have X, and (iii) feel entitled to X. Runciman (1966) added a fourth condition that individuals should (iv) see it as feasible that he should have X, whereas Gurr (1970) proposed exactly the opposite condition, maintaining that relative deprivation would arise when individuals see it not feasible to attain X. Bernstein and Crosby (1980) reconciled this discrepancy by introducing a temporal dimension. They found that relative deprivation is likely to be experienced when one feels that it was once feasible to attain X, and that it is not feasible to attain X in the future. Moreover, they also suggested that among all the preconditions identified, wanting is a necessary one.

Prior research has traditionally treated personal relative deprivation as a psychological response to unjust treatments experienced by specific individuals, such as women, ethnic minorities, or members of other disadvantaged social groups (e.g., Crosby, 1982; Tropp and Wright, 1999). Recently, Callan et al. (2008, 2011) have extended this line of research by viewing personal relative deprivation as an individual difference variable. They also induced it in laboratory settings by informing participants that they had less discretionary income than similar others. Given its great impact on individuals’ well-being and behaviors, it may be important to identify social factors besides unjust treatment that may lead to personal relative deprivation. We believe that materialistic cues may be one of such factors.

### Materialistic Cues and Personal Relative Deprivation

An adequate number of material possessions are necessary for anybody to survive. In a society which is increasingly materialistic, however, the meaning of money and material possessions goes far beyond necessities of life (e.g., Podoshen et al., 2011). Money and material possessions are associated with success, power, and social status, things which are inherently valued by human beings from the perspective of evolutionary psychology (Buss, 1995). Money and material possessions provide reliable protection for our fragile body and mind. They afford a sense of control, relieve our pain, and help us overcome the ultimate fear of death (e.g., Kasser and Sheldon, 2000; Zhou et al., 2009). In sum, money and material possessions serve many evolutionary and practical functions. Although excessive materialism has been linked to diminished well-being (see Dittmar et al., 2014, for a recent review), it is still very appealing to many people to acquire more and more material possessions.

We contend that materialistic cues would bring about personal relative deprivation. As many other goals, the aspiration for material acquisition may be inert most of the time. However, a materialistic environment can activate materialistic aspirations (Bauer et al., 2012; Kim, 2013). More importantly, material signs make the comparison between self and others painfully salient. It was noted by Karl Marx one and a half centuries ago that “A house may be large or small; as long as the surrounding houses are equally small, it satisfies all social demands for a dwelling. But let a palace arise beside the little house, and it shrinks from a little house into a hut” (Marx, 1847). We think that desirable yet unattainable material goods would trigger upward social comparison and remind people of their disadvantaged financial situations.

According to Bernstein and Crosby (1980), individuals would be more likely to experience relative deprivation if they believe that they are entitled to consumer goods unaffordable to them. In societies where there are legitimate, stable and widely accepted rules to determine the distribution of wealth, disadvantaged individuals may accept their own situation with little resentment. However, when the rules of wealth distribution are chaotic, and are seen as illegitimate or in a constant state of flux, individuals may feel undeserved when seeing themselves being worse-off than others (Ellemers et al., 1993; Feather, 2015). This is exactly the situation that faces the Chinese over the past decades (Griffin and Zhao, 1993; Li and Zhao, 2007). Therefore, we believe that the Chinese would feel resentful at the sight of unavailable wealth. They would think that they have not gotten what they deserve.

Some circumstantial evidence lends support to our hypothesis that materialistic cues would boost relative deprivation. For instance, Canache found that poor persons who reside in relatively well-off neighborhoods are more approval of political violence (Canache, 1996). Zhang et al. (2016) found that neighborhood socioeconomic status (SES) is positively associated with compulsive buying. People living in wealthy neighborhoods are frequently exposed to desirable consumer goods possessed by others which they themselves do not have. They may constantly experience relative deprivation owing to such upper social comparisons. Relative deprivation has been found to contribute to collective action against social injustice (e.g., Kawakami and Dion, 1993) and a preference for instant gratification (e.g., Callan et al., 2011). Therefore, it is conceivable that relative deprivation may be one common mechanism underlying the effects of neighborhood SES on individuals’ social attitudes and consumption behaviors.

In sum, we believe that materialistic cues satisfy at least four preconditions of relative deprivation: they represent things not attainable in the future for most people, stimulate “wanting,” encourage social comparison, and elicit feelings of entitlement.

### The Present Studies

We conducted three studies to investigate whether materialistic cues would boost personal relative deprivation, one in natural settings, and the other two in the laboratory. We hypothesized that being exposed to materialistic cues would increase individuals’ sense of relative deprivation. Moreover, we also explored the moderating roles of SES and trait materialism in this effect.

## Study 1

Materialistic cues are ubiquitous in our environment. In this study, we were inspired by Pyszczynski et al.’s (1996) research on mortality salience and examined the effect of incidental reminders of materialism in natural settings. We hypothesized that materialistic cues would lead to perceptions of relative deprivation. We also explored whether SES would moderate this effect. Due to their relatively better financial situation, individuals with higher SES may not feel as deprived as those with lower SES when exposed to materialistic cues.

### Method

#### Participants

Forty-seven men and 42 women participated in this survey. Their ages ranged from 15 to 65 years (*M* = 24.79, *SD* = 9.00). The brief survey was conducted in downtown Nanjing, the capital city of Jiangsu Province, China.

#### Procedure and Setting

Participants were interviewed 150 m before, directly in front of, or 150 m after the Prada store. The Prada store locates at the end of a series of luxury stores. It is a grand building covered with golden and silver striations. With all its salient signs and large display windows, it makes a perfect spot to cue materialism. Participants were interviewed either right in front of the Prada store (i.e., the materialistic-cue condition), or in front of an ordinary building without any sign (i.e., the two control conditions). Whereas those interviewed before the Prada store were on their way to the luxury stores, those interviewed after the Prada store were walking in the opposite direction, before they were stopped by the experimenters.

Pedestrians walking alone were stopped at the appropriate locations by two female experimenters and asked to participate in a brief survey conducted by the social psychology laboratory of Nanjing University. In the materialistic-cue condition, the experimenters positioned themselves so that participants faced one of the display windows of the Prada store during the interview. In the two control conditions, participants faced the ordinary building. They were not able to see the luxury stores or the signs during their interviews. Participants were asked the critical question “To what extent do you agree that you have got what you deserve compared to others?” Participants responded to this question on a 6-point scale ranging from 0 (*strongly disagree*) to 5 (*strongly agree*). We calculated a measure of relative deprivation by subtracting the answers from 6, so that higher scores indicate higher levels of relative deprivation. We then recorded participants’ age and SES. To assess SES, we asked participants to indicate their placement on a scale ranging from 1 to 10, where 1 represents the group with lowest levels of education and income in the society and 10 the highest. Participants were then given an opportunity to ask questions, and were thanked for participation.

### Results

No significant difference in SES was found among the three groups, *F*(2,86) = 1.70, *p* = 0.19 (**Table 1**). We then conducted a one-way ANOVA to determine whether materialistic cues influenced individuals’ perceptions of relative deprivation. The main effect of the survey location was significant, *F*(2,86) = 3.94, *p* = 0.023, $\eta_{p}^{2}$ = 0.084. A similar result was obtained with SES being controlled for, *F*(2,85) = 3.17, *p* = 0.047, $\eta_{p}^{2}$ = 0.069. Helmert comparisons showed that those interviewed right in front of the Prada store experienced higher levels of relative deprivation than those interviewed before or after the store, *p* = 0.023. The comparison between those surveyed in front of the Prada store and those before it yielded a significant result, *M*_D_ = 0.73, 95% CI = [0.15, 1.30], *p* = 0.014, Cohen’s *d* = 0.71. However, no significant difference was found between those surveyed in front of the Prada store and those after it, *p* = 0.126.

**Table 1:** Means (SDs) of the main variables as a function of experimental condition (Study 1).

	Before the luxury store	In front of the luxury store	After the luxury store
SES	5.13 (1.47)^a^	4.31 (1.77)^a^	4.55 (1.98)^a^
Relative deprivation	2.27 (1.00)^a^	3.07 (1.24)^b^	2.61 (1.02)^ab^

*SES, socioeconomic status; Values with different superscripts in the same line are significantly different from each other.*

We further examined whether participants’ SES would moderate the effect of materialistic cues on relative deprivation. A hierarchical regression was conducted with age, gender, SES, and survey location (dummy coded, 1 denotes in front of the Prada store and 0 denotes before or after the store) entered in the first block and the interaction between SES and survey location entered in the second block. The interaction term was non-significant, β = 0.01, *t* = 0.09, *p* = 0.93. Therefore, the effect of materialistic cues on relative deprivation seemed not to vary according to participants’ SES.

### Discussion

Generally, those interviewed right in front of a luxury store reported higher levels of relative deprivation than those interviewed without any luxurious signs in their immediate environment. However, the difference in relative deprivation between those interviewed in front of the luxury store and those who had just passed it did not reach significance. It was probably due to the fact that for those who had just come across a line of luxury stores (and maybe had even visited several of them), the visual impact of the material signs might still linger in their minds.

We expected that individuals with higher SES would be less influenced by materialistic cues than those with lower SES. However, this hypothesis was not supported. Material pursuits are virtually endless. For many people, no matter how wealthy they already are, they may still have financial aspirations that are not fulfilled. Hence, materialistic cues may invoke a sense of discontent regardless of individuals’ SES. It should be noted that participants’ subjective SES may have also been influenced by the situation they were interviewed. Indeed, those interviewed in front of the luxury store tended to report lower SES than those interviewed before or after the store, though the differences did not reach statistical significance. In the next study, we avoided this problem by assessing SES before the experimental manipulation. Study 1 assessed only the cognitive component of relative deprivation. However, emotional reactions such as anger and resentment are seen as an indispensable element of relative deprivation too (Smith et al., 2012). In Study 2, we also included measures of the affective component of relative deprivation.

## Study 2

In this study, we sought to replicate the findings from Study 1 with a different procedure. Specifically, we exposed participants to pictures of luxurious goods in laboratory, and examined whether such materialistic cues would have similar effects as the materialistic priming employed in Study 1. Moreover, we also included another potential moderator, trait materialism, in our investigation.

Trait materialism is a relatively stable individual difference variable characterized by revolving one’s life round acquiring material possessions and viewing material possessions as signs of success and happiness (Richins and Dawson, 1992). Trait materialism is linked to heightened materialistic longings. For those who are high (vs. low) on trait materialism, materialistic cues are more relevant to their personal aspirations. Thus, we expected that individuals scored higher on materialism would be more prone to relative deprivation when primed with materialistic cues than those lower on materialism.

### Method

#### Participants

Seventy-two graduate and undergraduate students (30 men, 42 women) participated in this study. Their mean age was 20.93 (*SD* = 1.90). Participants were recruited from a large subject pool. They received a small gift (worth around US$ 5) for their participation.

#### Procedure and Materials

Participants completed questionnaires measuring trait materialism and SES at least 1 week before the experiment.

The procedure of the experiment was adapted from Bauer et al. (2012). Specifically, participants were informed that the experiment was conducted to evaluate whether a number of pictures were suitable for future research on memory. They were seated in separated cubicles in our laboratory. Pictures were presented to them on computer screens one by one, each lasting for 2 s. Participants in the control group viewed 20 pictures of neutral objects (e.g., flowers, trees, groceries, and ordinary buildings), whereas participants in the materialistic-cue group viewed 12 pictures of luxury goods (including jewelry, wristwatches, fancy cars, and luxury houses) and 8 of neutral objects (e.g., flowers and trees). They were then presented with another set of 20 pictures, 10 new pictures and 10 from the pictures they had viewed, and asked to indicate whether they had seen the pictures before.

In a pilot study, 52 undergraduate students were asked to evaluate the pictures used in the study. Specifically, participants indicated how luxurious or expensive, attractive, and interesting the objects depicted in the pictures were. It was confirmed that pictures presented to the materialistic-cue group (*M* = 5.58, *SD* = 1.17, on a 7-point Likert scale) depicted objects more luxurious or expensive than those presented to the control group (*M* = 3.42, *SD* = 1.21), *t*(50) = 8.53, *p* < 0.001. However, the objects in the pictures were matched in attractiveness and interest, *ts*(50) < 1.10, *ps* > 0.30.

To buttress our cover story, after picture recognition, participants rated whether the pictures were difficult to memorize on a scale from 1 (*not at all*) to 7 (*very difficult*)^2^. They also indicated how pleasant and depressed they felt after viewing the pictures [from 1 (*not at all*) to 7 (*very much*)]. The two items (*r* = -0.44) were combined to form a positive affect index. Then, participants were asked to fill out a set of ostensibly unrelated questionnaires assessing their feelings at that time, which included our measure of state relative deprivation.

#### Measures

##### SES

We administered two measures of SES. First, participants completed the MacArthur Scale of subjective SES (e.g., Piff et al., 2010). In this measure, participants were shown a drawing of a ladder with 10 rungs, representing people with different levels of education, income, and occupation status. One denotes the lowest rung and 10 denotes the highest rung. Participants should select the rung they feel they belong to (*M* = 5.29, *SD* = 1.38). Moreover, as a measure of objective SES, participants also rated their parents’ highest levels of education completed and their annual household income. Education was coded into four categories: (i) did not finish high school, (ii) high school graduate or some college, (iii) college graduate, or (iv) postgraduate degree. Fathers’ median level of educational attainment was college graduation, and mothers’ median level of educational attainment was high school graduation or some college. Annual income was assessed using six categories: (i) below CNY 50,000, (ii) CNY 50,000-100,000, (iii) CNY 100,000-300,000, (iv) CNY 300,000-600,000, (v) CNY 600,000-1000,000, (vi) CNY 1000,000 and above. Participants had a median annual household income of between CNY 100,000 and 300,000.

The subjective (i.e., participants’ scores on the MacArthur Scale) and objective (i.e., the standardized and averaged parental educational attainment and annual household income, Cronbach’s α = 0.76) measures of SES correlated significantly (*r* = 0.58, *p* < 0.01), and were standardized and averaged to compute an overall measure of SES.

##### Trait materialism

Trait materialism was measured with the 18-item materialism scale developed by Richins and Dawson (1992). The materialism scale measures three components of materialism: *centrality* (i.e., the extent to which one centers his/her life around possessions and their acquisition; a sample item is “I enjoy spending money on things that aren’t practical”), *happiness* (i.e., the extent to which one view acquisition as a precondition of happiness; a sample item is “I’d be happier if I could afford to buy more things”), and *success* (i.e., the extent to which one judges people’s success on the basis of the possessions they accumulate; a sample item is “The things I own say a lot about how well I’m doing in life”). Participants responded to these items on a 7-point Likert scale, ranging from 1 (*Strongly disagree*) to 7 (*Strongly agree*). Cronbach’s α for this scale was 0.81.

##### Personal relative deprivation

The two items to assess personal relative deprivation were “Right now, I think I have got what I deserve compared to others” (reverse scored), and “Right now, I feel resentful and angry toward the situation that many people are better-off than me.” Participants responded to the items on a 7-point Likert scale, ranging from 1 (*Strongly disagree*) to 7 (*Strongly agree*). Answers on the two items were averaged (*r* = 0.31).

### Results

Descriptive statistics are shown in **Table 2**. No significant difference was found in terms of SES or trait materialism between the two groups, *ts*(70) < 1.71, *ps* > 0.09. However, participants in the materialistic-cue group reported lower levels of positive mood, *t*(70) = 2.74, *p* = 0.008, *M*_d_ = 0.70, 95% CI = [0.19, 1.21], *d* = 0.64, and higher levels of relative deprivation, *t*(70) = 3.20, *p* = 0.002, *M*_d_ = 0.69, 95% CI = [0.26, 1.11], *d* = 0.76, than those in the control group. The difference in relative deprivation between the two groups remained significant when SES, trait materialism, or both variables were controlled for^3^. It also remained significant when the effect of mood was partialled out, *F*(1,69) = 5.46, *p* = 0.022^4^.

**Table 2:** Means (SDs) of the measures as a function of experimental condition (Study 2).

	Materialistic-cue condition	Control condition
SES	-0.05 (0.84)^a^	0.04 (0.79)^a^
Trait materialism	3.91 (0.60)^a^	4.17 (0.66)^a^
Positive mood	5.04 (1.22)^a^	5.74 (0.94)^b^
Relative deprivation	3.20 (0.95)^a^	2.51 (0.87)^b^

*SES, socioeconomic status; Values with different superscripts in the same line are significantly different from each other.*

Two hierarchical regressions were performed to examine whether SES or trait materialism moderated the effect of materialistic cues on relative deprivation. In the regressions, the first block contained the main effects, and the second block included the interaction term. Both interactions were found to be non-significant; for the interaction between SES and experimental condition, β = -0.24, *t* = -0.67, *p* = 0.51, for the interaction between trait materialism and experimental condition, β = -0.09, *t* = -0.28, *p* = 0.79.

### Discussion

Participants who had just been exposed to materialistic cues reported higher levels of personal relative deprivation than those exposed to neutral scenes. Moreover, the discrepancy cannot be fully accounted for by the general emotional state they experienced. These results corroborated our hypothesis that materialistic cues would boost personal relative deprivation. Again, the effect of materialistic cues was independent of individuals’ SES.

Contrary to our prediction, we failed to find a significant moderating effect of trait materialism. In other words, materialistic cues evoked a sense of relative deprivation even among those who generally had low levels of materialistic aspirations. The visual impact of materialistic cues seemed to be powerful enough to temporarily override individuals’ chronic tendencies and to evoke inflated feelings of inadequacy and resentment in all. However, the validity of such conclusion may be questionable due to the small sample size involved. We conducted Study 3 to replicate the effect of materialistic cues on relative deprivation, and further examine the interaction between trait materialism and materialistic cues.

## Study 3

Study 3 was a replication of Study 2, with a larger sample size and a more stringent measure of relative deprivation.

### Method

#### Participants

One hundred and twenty graduate and undergraduate students (35 men, 85 women) participated in this study. The mean age was 20.70 (*SD* = 1.58). Participants were recruited from a large subject pool. They were paid CNY30 (around US$ 5) for their participation.

#### Materials, Procedure, and Measures

The materials, procedure, and measures involved in this study were all the same as those used in Study 2, except that relative deprivation was measured with the personal relative deprivation scale (Callan et al., 2008). The scale contains four items assessing both cognitive and emotional components of personal relative deprivation. A sample item is “When I think about what I have compared to others, I feel deprived.” Participants rated the extent to which they agreed with each item on a 7-point Likert scale ranging from 1 (*strongly disagree*) to 7 (*strongly agree*). Cronbach’s α for this scale was 0.64.

### Results

**Table 3** presents the descriptive statistics. No significant difference was found in SES, trait materialism, or positive mood between the two groups, *ts*(118) < 1.07, *ps* > 0.29. However, as predicted, participants in the control group reported lower levels of relative deprivation than those in the materialistic-cue group, *t*(118) = 2.63, *p* = 0.010, *M*_d_ = 0.40, 95% CI = [0.10, 0.69], *d* = 0.48. The difference in relative deprivation between the two groups remained significant when SES, trait materialism, and mood were controlled for, *F*(1,115) = 5.63, *p* = 0.019.

**Table 3:** Means (SDs) of the measures as a function of experimental condition (Study 3).

	Materialistic-cue condition	Control condition
SES	-0.01 (0.98)^a^	0.01 (1.03)^a^
Trait materialism	4.10 (0.90)^a^	4.09 (0.92)^a^
Positive mood	5.17 (1.04)^a^	5.38 (1.10)^a^
Relative deprivation	3.19 (0.73)^a^	2.80 (0.91)^b^

*SES, socioeconomic status; Values with different superscripts in the same line are significantly different from each other.*

Results from hierarchical regressions showed that neither SES nor trait materialism had significant moderating effects on the association between materialistic cues and relative deprivation (for the interaction between SES and experimental condition, β = -0.02, *t* = -0.19, *p* = 0.85; for the interaction between trait materialism and experimental condition, β = -0.07, *t* = -0.71, *p* = 0.48).

## General Discussion

In the current studies, we found that mere exposure to materialistic cues (e.g., a luxury store, pictures of luxury goods) increased individuals’ sense of relative deprivation. After viewing material signs, individuals are more likely to believe that they are unjustly worse-off than others, and feel resentful about the situation. This effect was not significantly moderated by either SES or trait materialism.

Although abundant research has been accumulated on the negative association between materialism and well-being (see Dittmar et al., 2014, for a review), our work is the first to show that being exposed to materialistic cues contributes to a psychological state that is usually tied to social comparison. It implies that materialistic cues stand not only for signs of success and popularity, but also for a reference point (though unrealistic) to gauge one’s own placement in a society. Our research also provides the first evidence that social cues which bear no strong personal relevance can lead to a sense of relative deprivation. It implies that to experience relative deprivation, individuals do not actually need to undergo unfair treatment or to be put at disadvantage. A few materialistic signs are already sufficient to activate the sense of relative deprivation.

Previous research from our laboratory has shown that personal relative deprivation increases materialism (Zhang et al., 2015). The current studies, however, show that the reverse is also true. The logical consequence is an “upward spiral” of reciprocal causality between materialism and personal relative deprivation. Relative deprivation, as aroused by materialistic cues and thoughts, leads to even higher levels of materialistic aspirations. Moreover, just like materialism, personal relative deprivation has also been found to contribute to certain negative psychological states, such as diminished well-being (e.g., Hafer and Olson, 1993; Eibner et al., 2004) and self-control deficits (Callan et al., 2011). Future research on the mechanisms of materialism may incorporate the line of research on relative deprivation, and study materialism from both the personal level and the perspective of social comparison.

Why, then, do materialistic cues induce a sense of relative deprivation? We believe that the key to this phenomenon lies in the interplay between humans’ innate propensity and prevailing social norms. Human beings have an inherent preference for material possessions, and a materialistic culture legitimates and encourages materialistic aspirations. In a society where people are disproportionately judged by their material possessions, material goods do not only mean life necessities, but also signal social acceptance and status (Saxe and Haushofer, 2008; Zhou and Gao, 2011). It may pose a great threat to individuals’ self-image when being exposed to expensive goods they cannot afford. Consequently, individuals may react defensively to such cues. They may think that they are unjustly deprived and feel resentful. Such way of thinking should be readily justified by rapid economic development, increasing inequality, and ever-changing distribution rules in the Chinese society.

This analysis alludes to potential cultural differences. Relative deprivation may not be increased at the sight of materialistic cues in societies where materialism is not so enthusiastically endorsed, or where wealth is more fairly distributed. However, given the irresistible attractiveness of material possessions to the human mind, we believe that the effect of materialism on relative deprivation would also be found in many other societies.

We did not find the hypothesized moderating effect of trait materialism. One explanation may be that materialistic situations are powerful enough to override this individual difference. Therefore, even individuals who generally do not focus their attention on material possessions may be unable to resist the lure of luxury goods. The effect of materialistic cues on relative deprivation was not moderated by participants’ SES either. This may be due to the fact that we used material goods that are highly desirable but beyond what most people can afford. Therefore, those with relatively high SES would still feel relative deprived when exposed to these goods. Future studies may investigate whether the effect of materialistic cues on relative deprivation would hold in more aﬄuent societies. Future studies may also examine whether individual differences in power and narcissism, variables that are tied to individuals’ sense of entitlement, would moderate the effect of materialistic cues on relative deprivation (e.g., Raskin and Terry, 1988; Sawaoka et al., 2015).

We investigated only the instant effect of materialistic cues on personal relative deprivation. It remains open whether the effect of materialistic cues would retain if people are constantly involved in materialistic environments. We think two opposite tendencies may be equally possible. First, individuals may be habitualized to materialistic cues. The level of relative deprivation they experience may return to baseline or remain constant after reaching a certain point. As an alternative possibility, the effect of materialistic on relative deprivation may be accumulated, leading to deleterious consequences such as depression, interpersonal conflict, or acts of sabotage. Longitudinal studies conducted in natural settings may provide an answer to this question. If the latter were the case, the society should find ways to prevent such consequences, such as promoting fair distribution of social wealth, reducing the income gap, cutting down advertising expenditures, and inhibiting conspicuous consumption.

There are several limitations in the current studies. First, although the measures of relative deprivation used in Studies 1 and 2 have face validity, reliability and construct validity of these items need to be established. We used an existing scale to measure relative deprivation in Study 3. However, the internal consistency was lower than optimal. Nevertheless, we believe that the consistent results across the three studies have provided substantial support for our hypothesis. Second, most of the participants in the studies were young. We don’t know whether similar effects would be obtained among older samples. Given the fluctuating importance of acquiring material possessions across the life span, we suspect that the effect of materialistic messages may show an inverted-U shaped association with age. Third, one reason why we did not find significant moderating effect of SES might be that participants involved in our studies had low-to-medium SES on average. Those who have extremely high SES may not experience relative deprivation when seeing materialistic cues. On the contrary, they may even feel satisfied and proud. Fourth, the current studies focused solely on relative deprivation, a subjective cognitive and emotional state. It is worthwhile to examine potential behavioral consequences, such as gambling tendency and compulsive buying. Future studies may investigate the adverse effects of relative deprivation as induced by materialistic cues and find ways to alleviate them.

## Conclusion

We observed in three studies that after viewing materialistic cues, participants experienced heightened levels of relative deprivation. These studies broaden our understanding of the antecedents of relative deprivation, and shed light on the mutual reinforcement of materialism and relative deprivation.

## Ethics Statement

This research is approved by the Institutional Review Board of Nanjing University.

## Author Contributions

HZ designed the study and drafted the paper. WZ did the majority work on data collection and data analysis.

## Conflict of Interest Statement

The authors declare that the research was conducted in the absence of any commercial or financial relationships that could be construed as a potential conflict of interest.

## Footnotes

1 We focus our discussion on personal relative deprivation which is supposed to be an outcome of interpersonal comparisons, rather than group relative deprivation which involves comparisons between groups (Runciman, 1966).

2 No significant difference was found regarding this variable between the two groups, *t*(70) = 0.93, *p* = 0.36.

3 When SES was controlled for, *F*(1,69) = 9.78, *p* = 0.003. When trait materialism was controlled for, *F*(1,69) = 9.72, *p* = 0.003. When both variables were controlled for, *F*(1,68) = 9.65, *p* = 0.003.

4 There were no significant effects involving either gender or age in all three studies.
